# Supplementary material for: FET family fusion oncoproteins target the SWI/SNF chromatin remodeling complex
Source: EMBO Rep. 2019 Apr 8;20(5):e45766. doi: 10.15252/embr.201845766 (PMC6500973; doi:10.15252/embr.201845766)
Supplement: Supplementary file 1 — Expanded View Figures PDF [file EMBR-20-e45766-s001.pdf]

Expanded View Figures

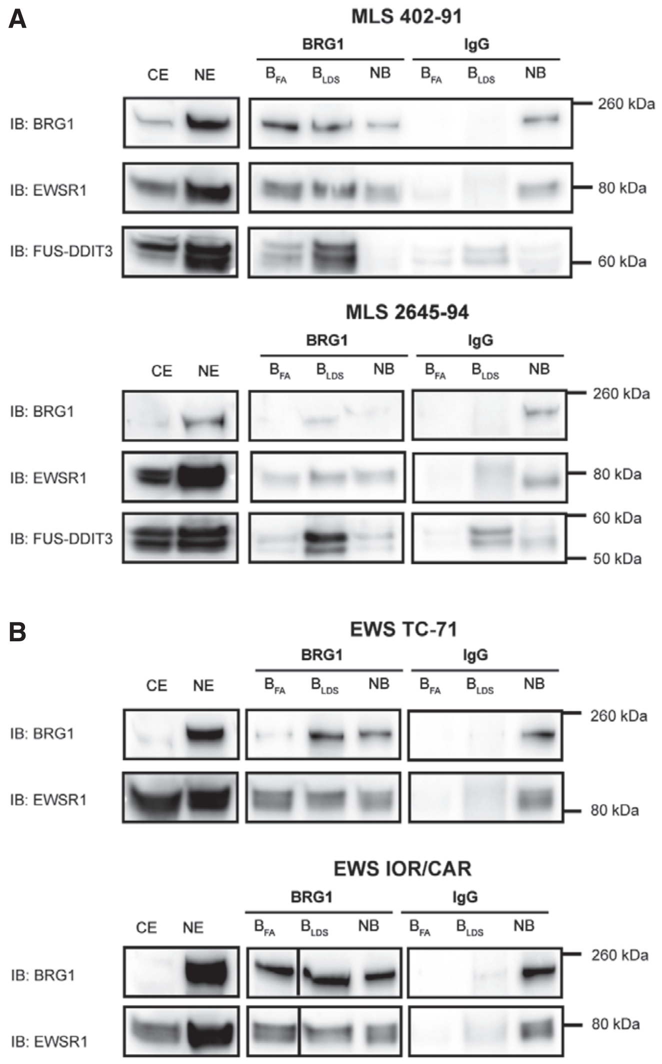

Figure EV1. BRG1 Co-IP of sarcoma cell lines eluted with formic acid.

A Immunoblot analysis (IB) of proteins co-immunoprecipitated with BRG1. Detection with antibodies against BRG1, EWSR1, and FUS-DDIT3 in MLS cell lines 402-91 and 2645-94.

B Immunoblot analysis (IB) of proteins co-immunoprecipitated with BRG1. Detection with antibodies against and BRG1 and EWSR1 in EWS cell lines TC-71 and IOR/CAR. CE: cytoplasmic extract, NE: nuclear extract, B_{FA}: bound proteins eluted with 1% formic acid, B_{LDS}: bound proteins eluted with LDS sample buffer, NB: non-bound proteins, not captured by the antibody.

Source data are available online for this figure.

 © 2019 The Authors

Figure EV2. Histone H3 modification analysis.

A Immunoblot analysis (IB) of histone modifications in replicate R2 of stably transfected HT1080 cell lines expressing EGFP, FUS-DDIT3-EGFP, or EWSR1-FLI1-EGFP. Antibodies against H3K27Ac, H3K27me3, H3K4me3, and histone loading control H4 for detection of histone modifications and antibodies against EZH2 and loading control GAPDH to evaluate catalytic PRC2 amount. Immunoblot analysis with GFP antibody is shown to verify expression of FET fusion oncoproteins and EGFP. Stable replicate R1 is visualized in Fig 4A.

B Immunoblot analysis (IB) of HT1080, and HT1080 transiently expressing FUS-DDIT3-EGFP or EWSR1-FLI1-EGFP (samples harvested after 24 or 48 h transient transfection) with antibodies against H3K27Ac, H3K27me3, H3K4me3, and histone loading control H4 for detection of histone modifications and antibodies against EZH2 and loading control GAPDH to evaluate catalytic PRC2 amount. Immunoblot analysis with GFP antibody is shown to verify expression of FET fusion proteins.

C, D Immunoblot analysis (IB) of HT1080 and HT1080 FUS-DDIT3-EGFP (C) or HT1080 EWSR1-FLI1-EGFP (D) (both stable expression) with antibodies against H3K27me3, H3K4me3, and histone loading control H4. Immunoblot analysis with GFP or FLI1 antibody is shown to verify expression of FET fusion protein and normal FLI1. Note that endogenous FLI1 around 50 kDa is expressed in HT1080 cell lines.

E Immunoblot analysis (IB) of extracts from untreated EWS TC-71, cells treated with 5 μM tazemetostat for 72 h and DMSO-control. Detection using antibodies against H3K27me3, H3K4me3, and histone loading control H4 showed substantial decrease of H3K27me3 after inhibition of EZH2 with tazemetostat.

F Diagrams showing amount, quantified from immunoblots in Fig 4A (R1) or panel (A) (R2), of H3K27me3, H3K4me3, and H3K27Ac (in relation to corresponding histone loading control H4), ratio H3K27me3/H3K4me3 as well as amount of EZH2 compared to loading control GAPDH for HT1080 EGFP, HT1080 FUS-DDIT3-EGFP, and HT1080 EWSR1-FLI1-EGFP normalized to parental HT1080. Each diagram panel shows data from one experiment.

G Diagrams showing amount, quantified from immunoblots in panel (B), of H3K27me3, H3K4me3, and H3K27Ac (in relation to corresponding histone loading control H4), ratio H3K27me3/H3K4me3 as well as amount of EZH2 compared to loading control GAPDH for HT1080 FUS-DDIT3-EGFP and HT1080 EWSR1-FLI1-EGFP normalized to parental HT1080. Samples were harvested after 24-h or 48-h transient transfection. Each diagram panel shows data from one experiment.

H, I Diagram showing amount of H3K27me3 and H3K4me3 (in relation to corresponding histone loading control H4) as well as ratio H3K27me3/H3K4me3 for HT1080 FUS-DDIT3-EGFP (H) quantified from panel (C) or HT1080 EWSR1-FLI1-EGFP (I) quantified from panel (D) (both stable expression) normalized to parental HT1080. Each diagram panel shows data from one experiment.

J Diagram showing amount, quantified from immunoblots in panel (E), of H3K27me3 and H3K4me3 (in relation to corresponding histone loading control H4) as well as ratio H3K27me3/H3K4me3 for untreated EWS TC-71, cells treated with 5 μM tazemetostat for 72 h and DMSO-control, normalized to the untreated control. Data from one experiment.

Source data are available online for this figure.

© 2019 The Authors

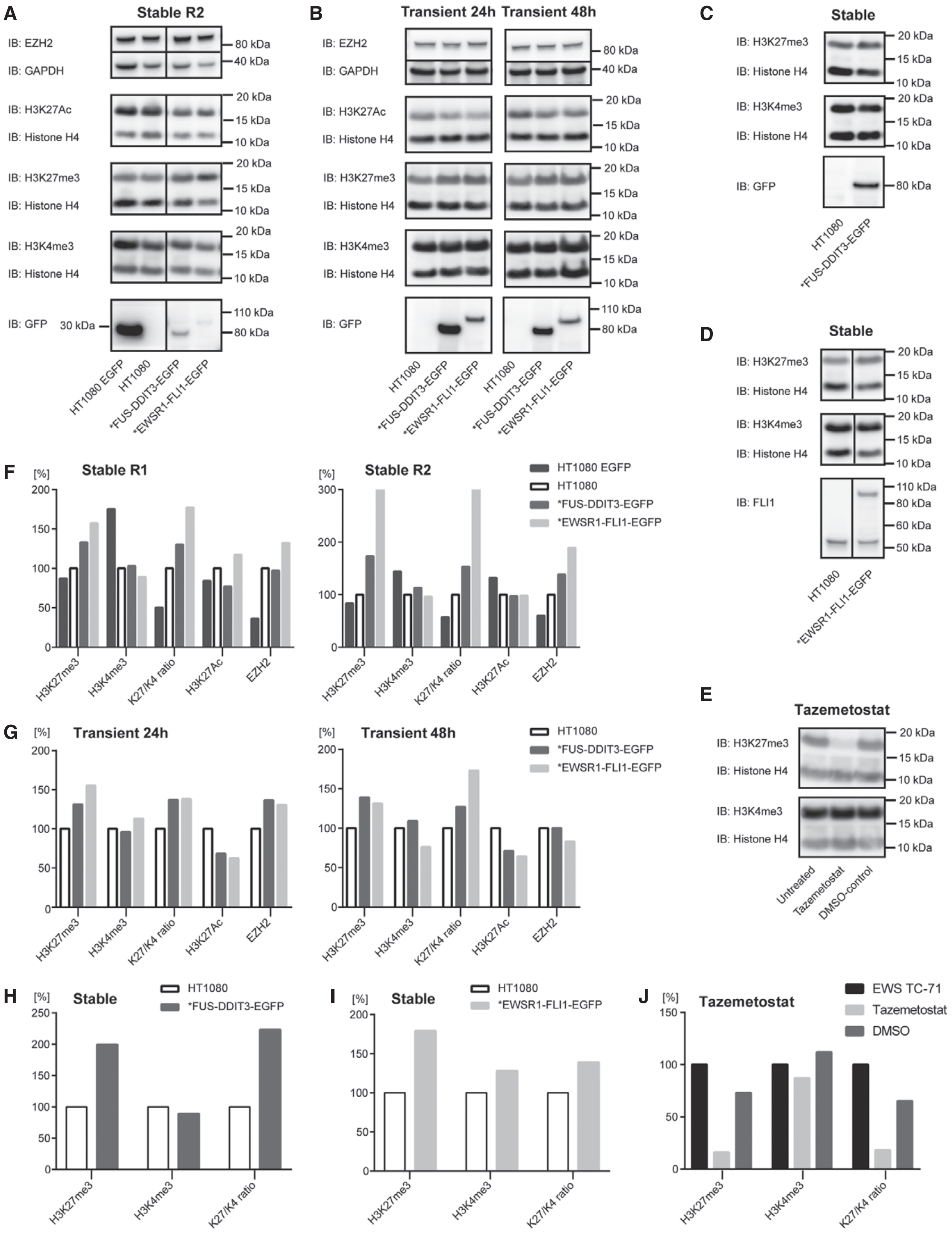

Figure EV2.

 © 2019 The Authors

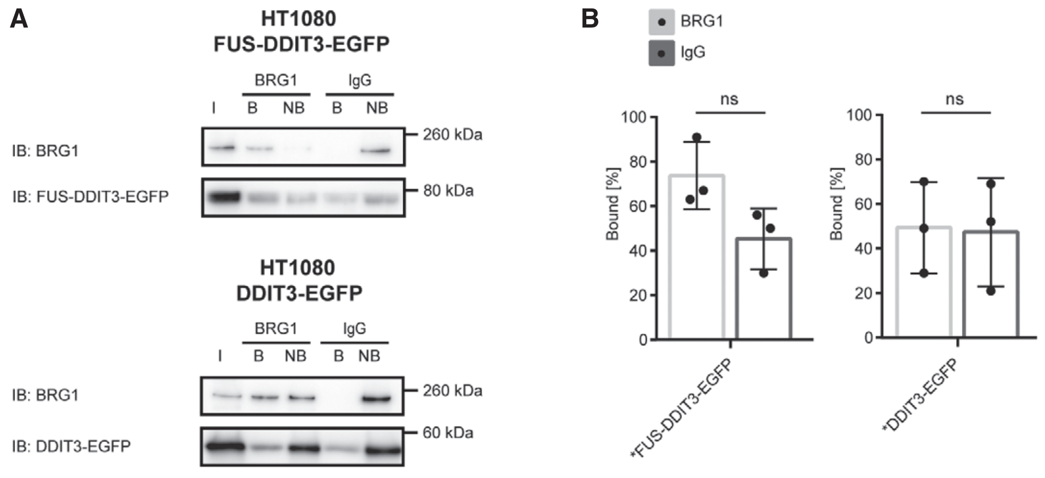

Figure EV3. BRG1 Co-IP to evaluate DDIT3 as potential binding partner.

A Immunoblot analysis (IB) of GFP-tagged proteins co-immunoprecipitated with BRG1. Detection with antibodies against BRG1 and DDIT3 in HT1080 cell lines with stable expression of FUS-DDIT3-EGFP or DDIT3-EGFP. In order to directly quantify the fraction of bound and non-bound protein, relative amounts of protein for each IP-sample were loaded on the gel, with consideration taken for dilutions during the immunoprecipitation procedure. I: input of nuclear extract, B: bound proteins, NB: proteins not bound. One representative immunoblot is shown. Immunoblots from all replicates are shown in the source data.

B Graphs showing the percentage of bound to total (bound + non-bound) signal intensities from immunoblots for DDIT3 in HT1080 FUS-DDIT3-EGFP or DDIT3-EGFP. The specific interaction (BRG1) is compared to the non-specific interaction (negative control IgG). Mean ± SEM is shown with individual replicates indicated by circles, n = 3. Student's t-test, ns = not significant. Original data for all quantifications, including P-values, are shown as source data.

Source data are available online for this figure.

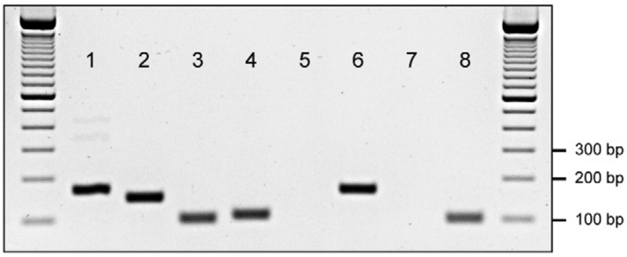

	Cell line	Fusion	Assay	Expected size
1	MLS 2645-94	FUS-DDIT3 Type II	FUS-DDIT3 Brkp2	167 bp
2	MLS 402-91	FUS-DDIT3 Type I	FUS-DDIT3 Brkp1	147 bp
3	EWS TC-71	EWSR1-FLI1 Type I	EWSR1(ex7)-FLI1(ex6)	99 bp
4	EWS IOR/CAR	EWSR1-ERG	EWSR1(ex7)-ERG	105 bp
5	HT1080	-	FUS-DDIT3 Brkp2	-
6	HT1080 FUS-DDIT3-EGFP	FUS-DDIT3 Type II	FUS-DDIT3 Brkp2	167 bp
7	HT1080	-	EWSR1(ex7)-FLI1(ex6)	-
8	HT1080 EWSR1-FLI1-EGFP	EWSR1-FLI1 Type I	EWSR1(ex7)-FLI1(ex6)	99 bp

Assay	Forward Primer	Reverse Primer
FUS-DDIT3 Brkp2	AGCAGAACCAGTACAACAGC	CCCGAAGGAGAAAGGCAATG
FUS-DDIT3 Brkp1	GACCGTGGTGGCTTCAATAA	CAGTGTCCCGAAGGAGAAA
EWSR1(ex7)-FLI1(ex6)	TCCTACAGCCAAGCTCCAAGTCAATA	ATTGCCCCAAGCTCCTCTTCTGAC
EWSR1(ex7)-ERG	TCCTACAGCCAAGCTCCAAGTCAATA	GCCGTGACCGGTCCAGGCT

Figure EV4. Cell line verification.
Verification of cell lines by RT–PCR over fusion breakpoints (brkp) of MLS 2645-94 (FUS-DDIT3 type II), MLS 402-91 (FUS-DDIT3 type I), EWS TC-71 (EWSR1-FLI1 type I), EWS IOR/CAR (EWSR1-ERG), HT1080 FUS-DDIT3-EGFP (FUS-DDIT3 type II), and HT1080 EWSR1-FLI1-EGFP (EWSR1-FLI1 type I): list of cell line, fusion type, assay used (forward and reverse primer) and expected product size indicated.

 © 2019 The Authors
