# Supplementary material for: FET family fusion oncoproteins target the SWI/SNF chromatin remodeling complex
Source: EMBO Rep. 2019 Apr 8;20(5):e45766. doi: 10.15252/embr.201845766 (PMC6500973; doi:10.15252/embr.201845766)

Figure EV1: MLS 402-91

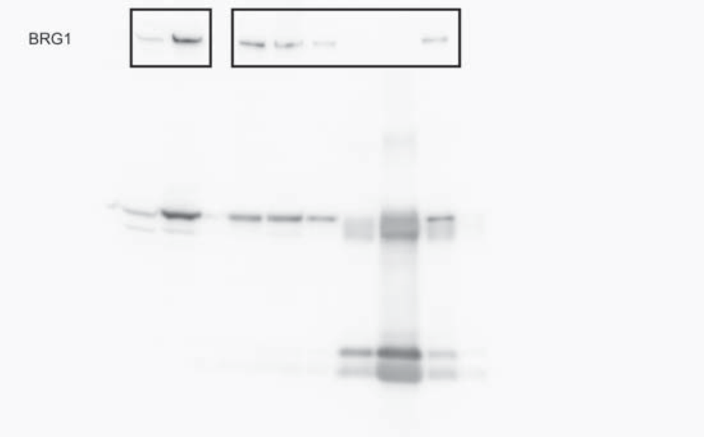

Figure EV1: MLS 402-91

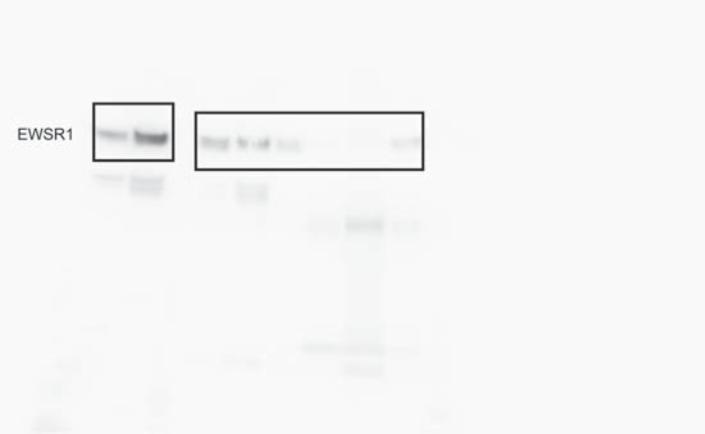

Figure EV1: MLS 402-91

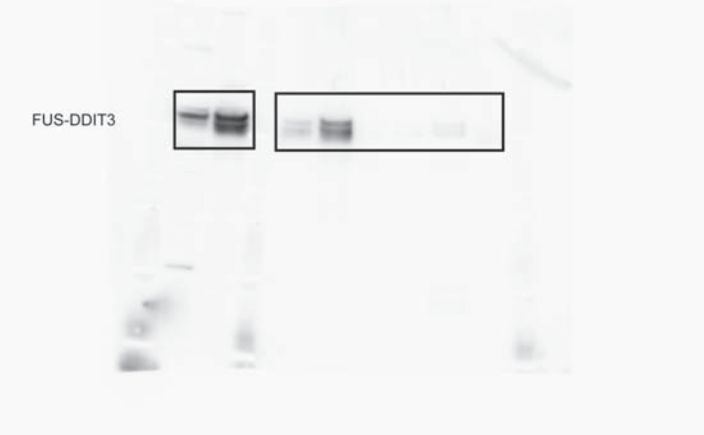

Figure EV1: MLS 2645-94

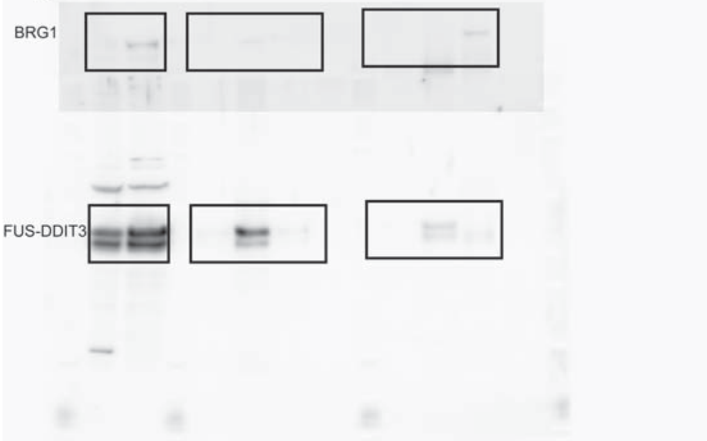

Figure EV1: MLS 2645-94

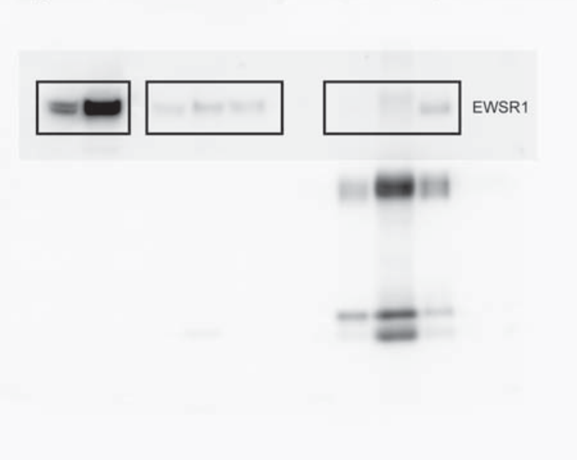

Figure EV1: EWS IOR/CAR

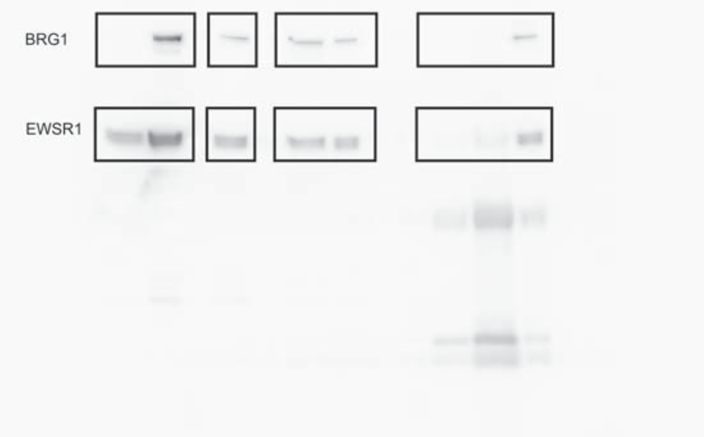

Figure EV1: EWS TC-71

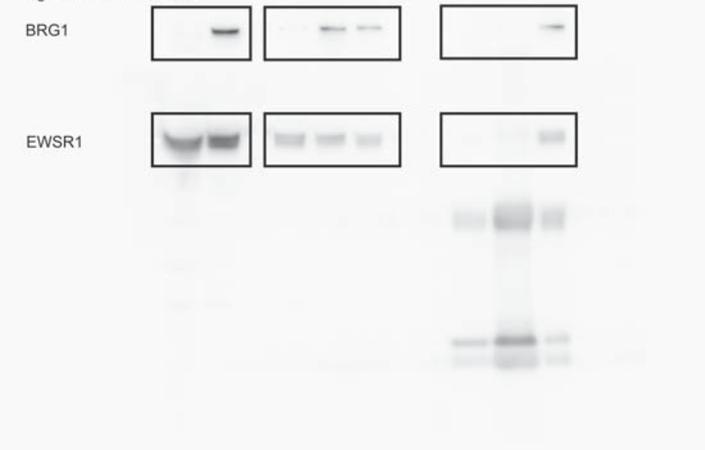

Supplement: Supplementary file 5 — Source Data for Expanded View [file EMBR-20-e45766-s009.zip › SourceDataForExpandedView/EMBOR-2018-45766_SourceDataForFigureEV1.pdf]
