# Supplementary figures and images for: FET family fusion oncoproteins target the SWI/SNF chromatin remodeling complex
Source: EMBO Rep. 2019 Apr 8;20(5):e45766. doi: 10.15252/embr.201845766 (PMC6500973; doi:10.15252/embr.201845766)

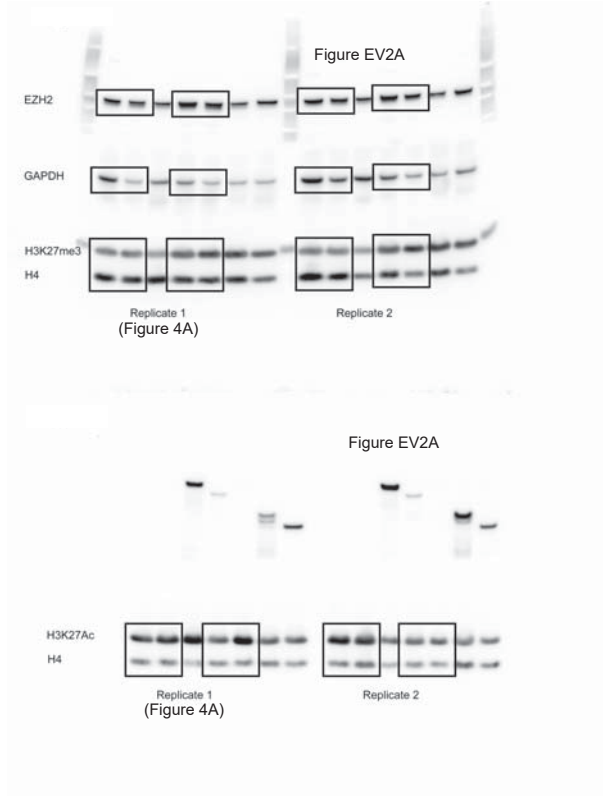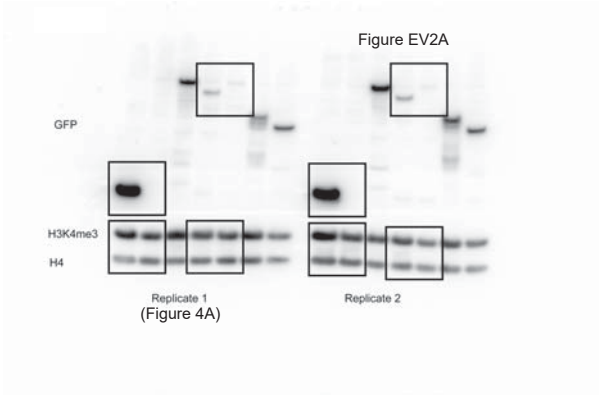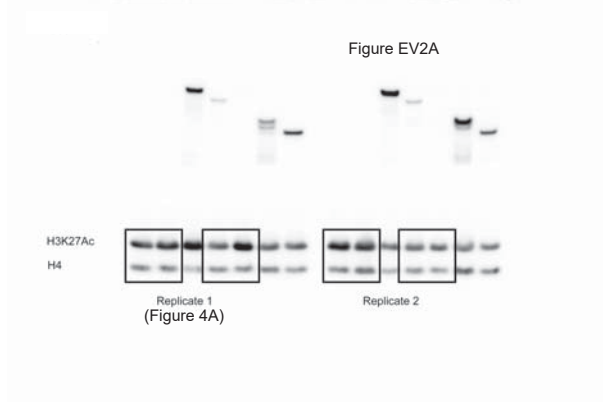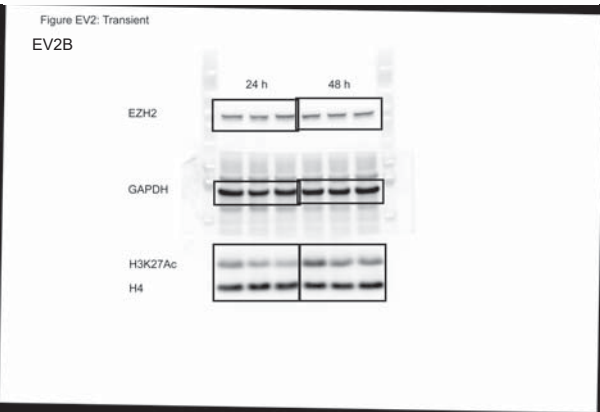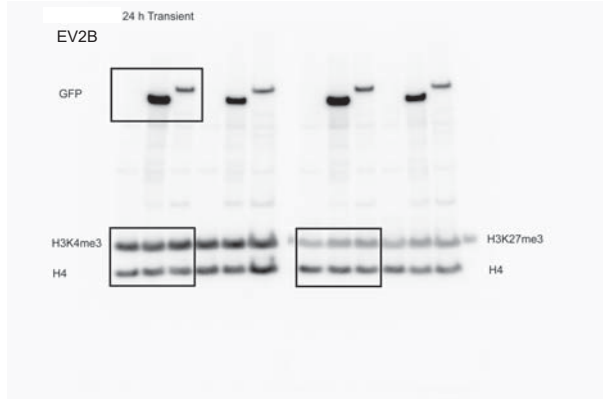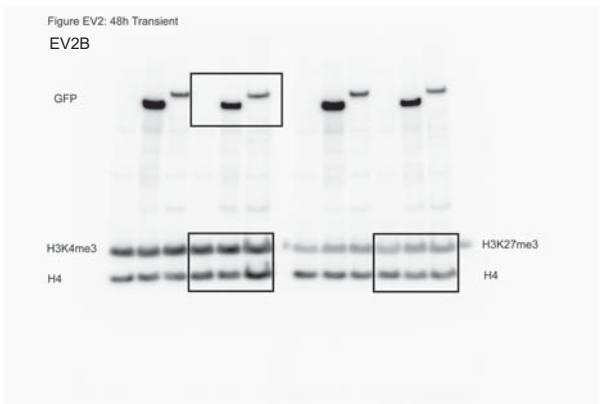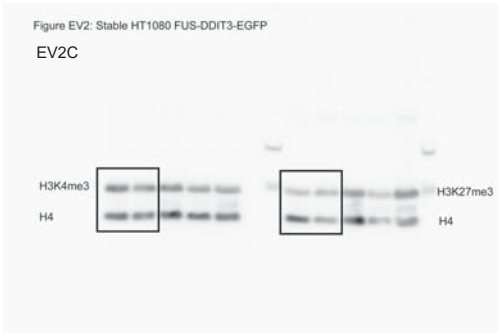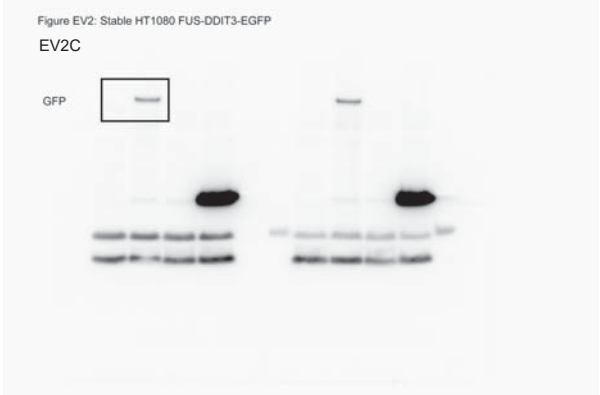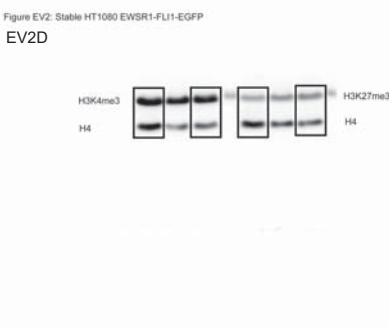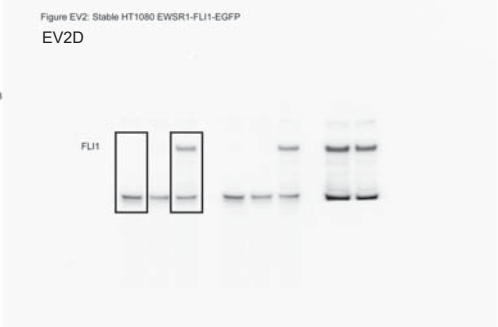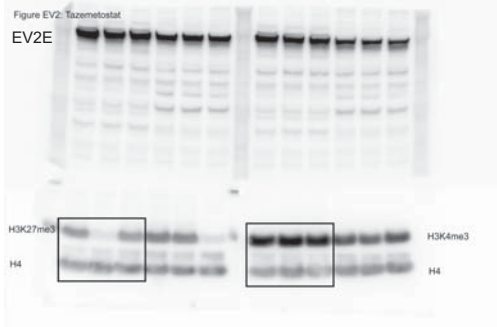

Supplement: Supplementary file 5 — Source Data for Expanded View [file EMBR-20-e45766-s009.zip › SourceDataForExpandedView/EMBOR-2018-45766_SourceDataForFigureEV2/EMBOR-2018-45766_SourceDataForFigureEV2A-E.pdf]

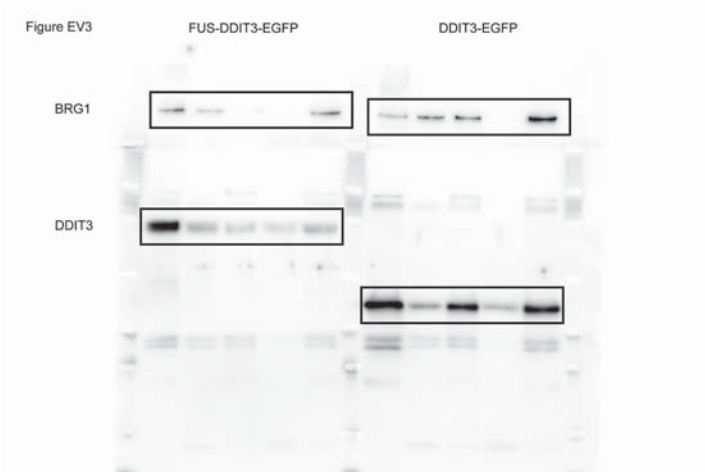

Figure EV3 Replicates

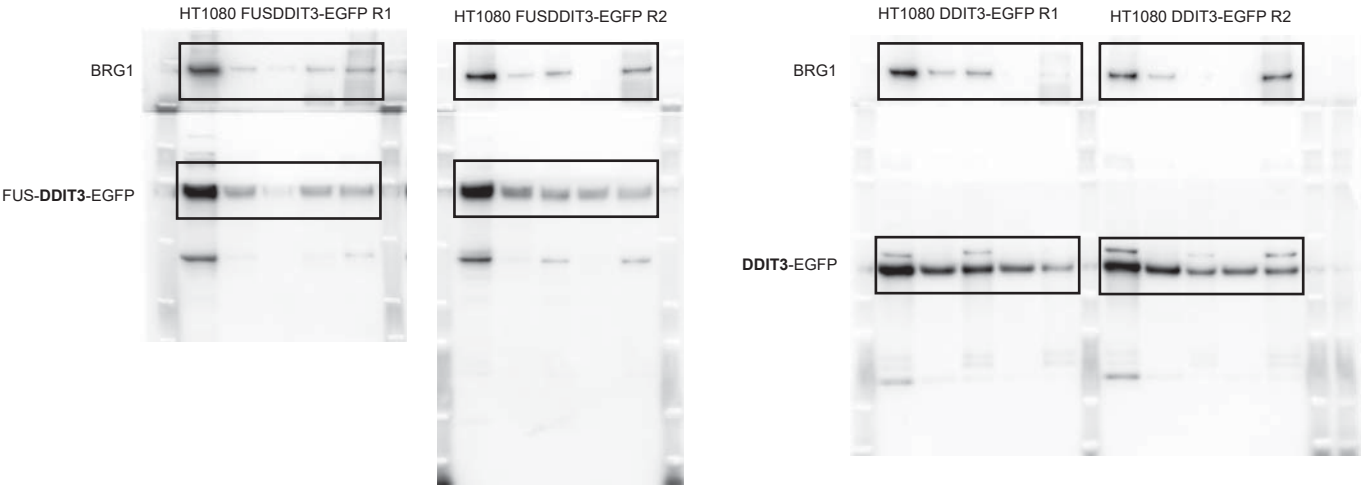

Supplement: Supplementary file 5 — Source Data for Expanded View [file EMBR-20-e45766-s009.zip › SourceDataForExpandedView/EMBOR-2018-45766_SourceDataForFigureEV3/EMBOR-2018-45766_SourceDataForFigureEV3A.pdf]

Figure 1D: ARID1A

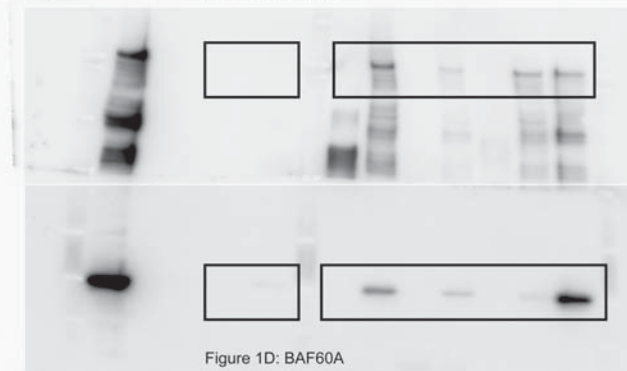

Figure 1D: BAF60A

Figure 1D: BRG1

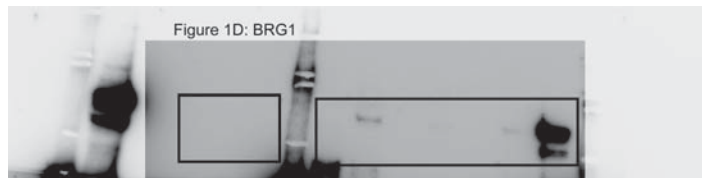

Supplement: Supplementary file 7 — Source Data for Figure 1 [file EMBR-20-e45766-s005.pdf]

Figure 2B

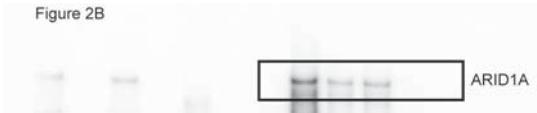

Figure 2B

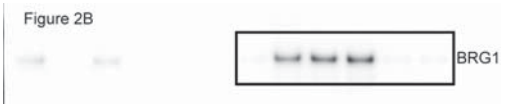

Figure 2C

ARID1A

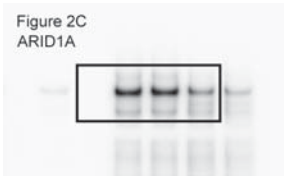

Figure 2C

BRG1

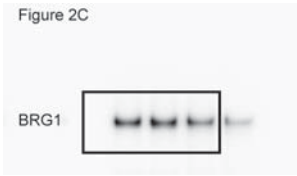

Figure 2D

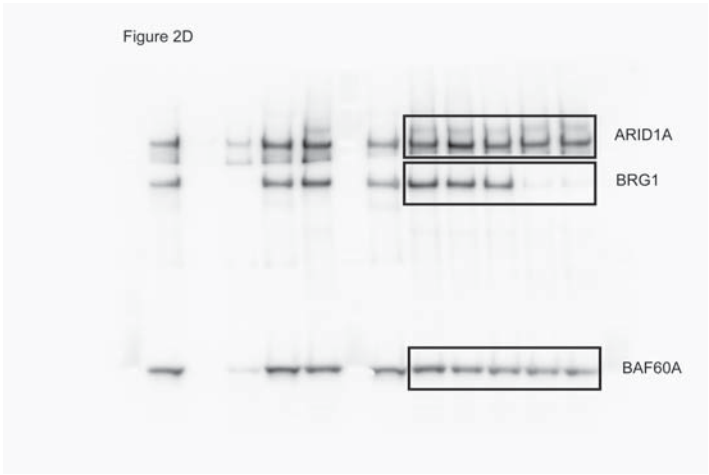

Figure 2D

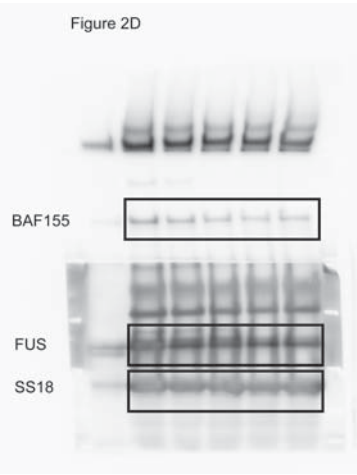

Figure 2D

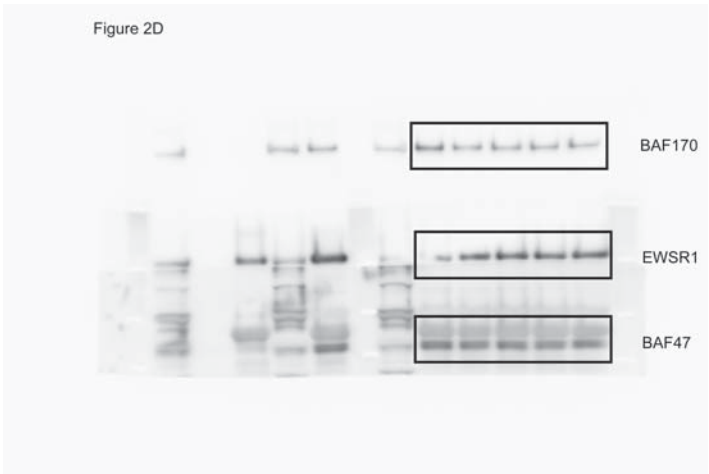

Supplement: Supplementary file 8 — Source Data for Figure 2 [file EMBR-20-e45766-s006.pdf]

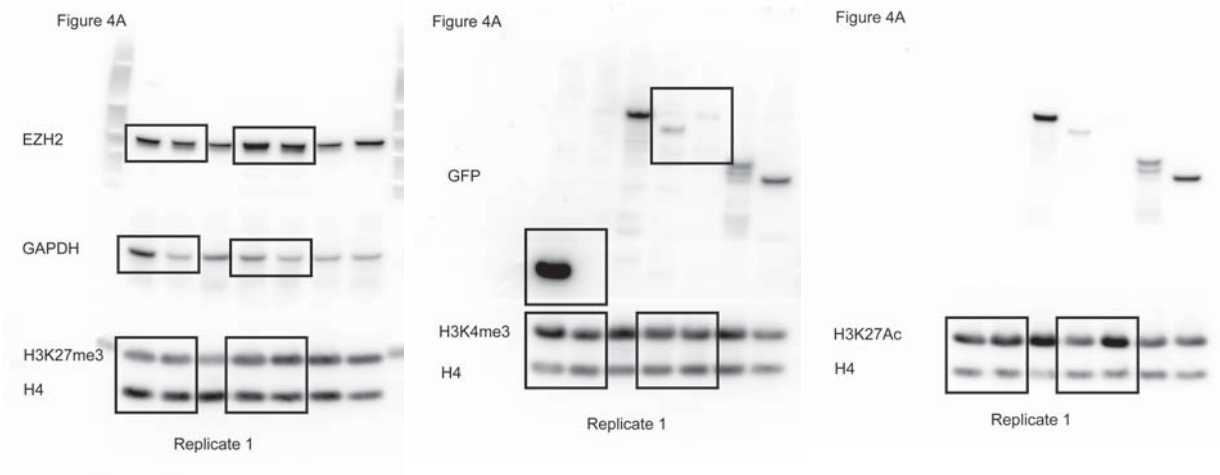

Supplement: Supplementary file 10 — Source Data for Figure 4 [file EMBR-20-e45766-s008.zip › EMBOR-2018-45766_SourceDataForFigure4/EMBOR-2018-45766_SourceDataForFigure4A.pdf]
