# Supplementary material for: FET family fusion oncoproteins target the SWI/SNF chromatin remodeling complex
Source: EMBO Rep. 2019 Apr 8;20(5):e45766. doi: 10.15252/embr.201845766 (PMC6500973; doi:10.15252/embr.201845766)

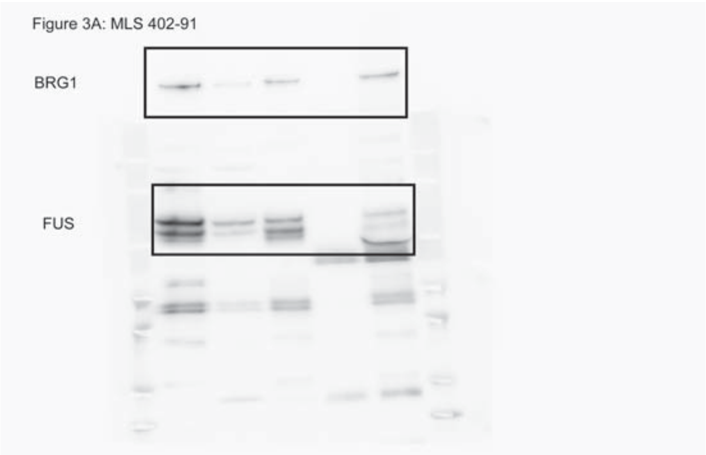
Figure 3A: MLS 402-91
BRG1
FUS

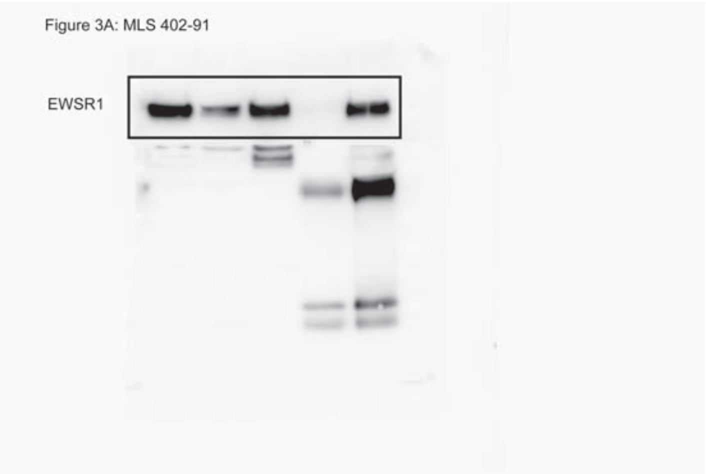
Figure 3A: MLS 402-91
EWSR1

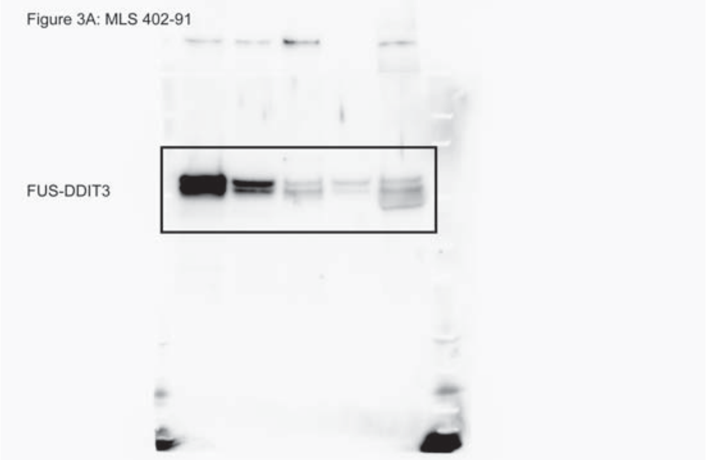
Figure 3A: MLS 402-91
FUS-DDIT3

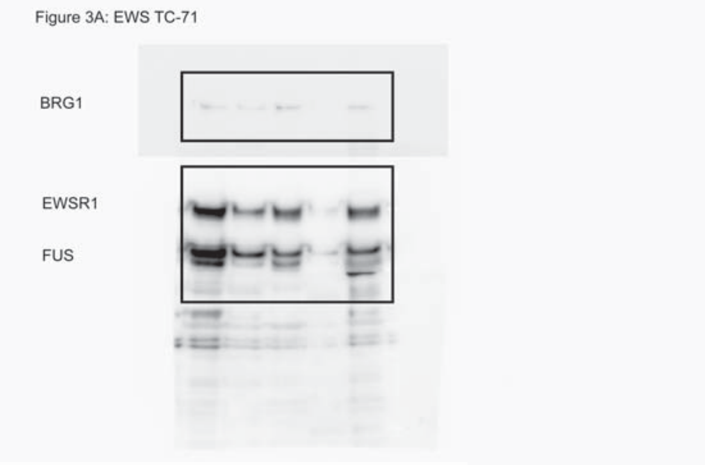
Figure 3A: EWS TC-71
BRG1
EWSR1
FUS

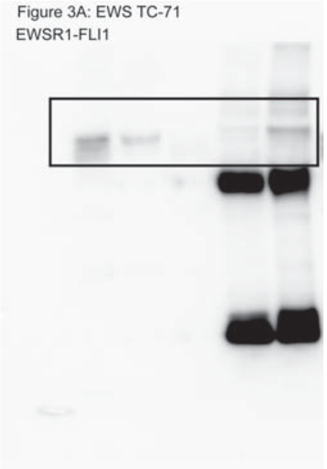
Figure 3A: EWS TC-71
EWSR1-FLI1

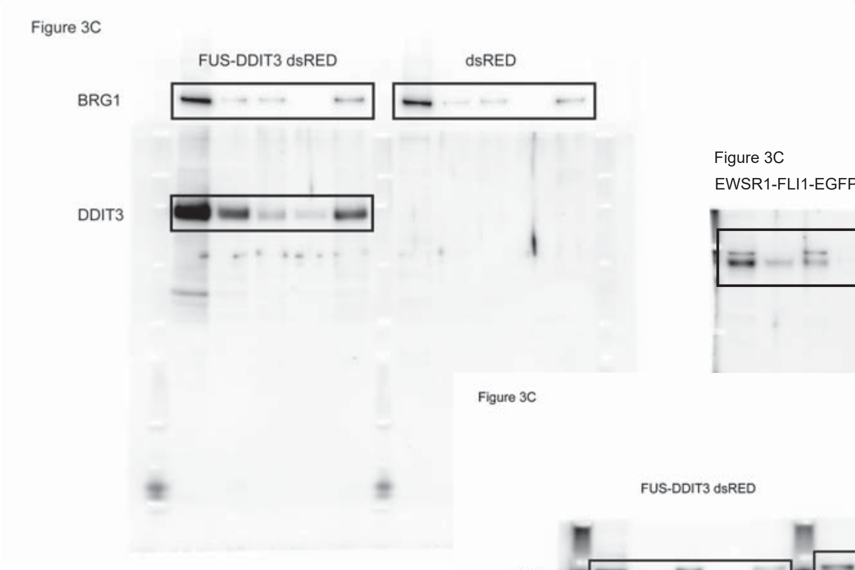
Figure 3C
FUS-DDIT3 dsRED
dsRED
BRG1
DDIT3

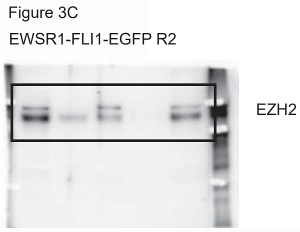
Figure 3C
EWSR1-FLI1-EGFP R2
EZH2

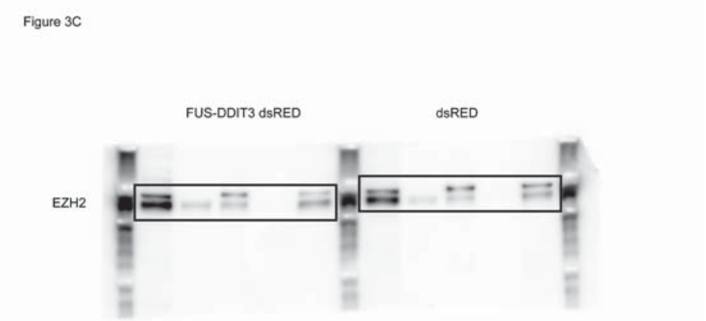
Figure 3C
FUS-DDIT3 dsRED
dsRED
EZH2

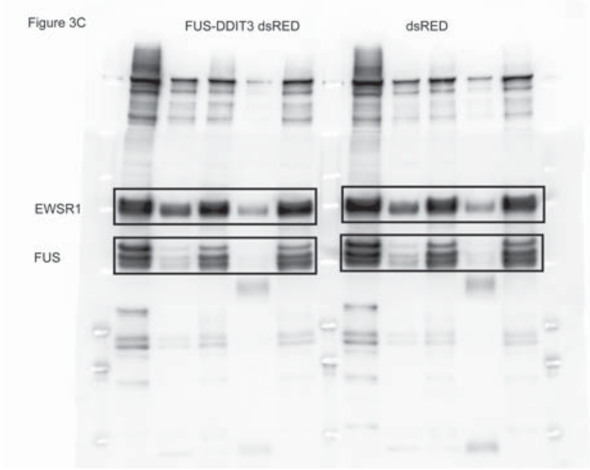
Figure 3C
FUS-DDIT3 dsRED
dsRED
EWSR1
FUS

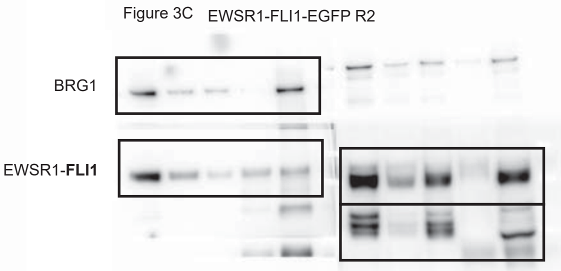
Figure 3C
EWSR1-FLI1-EGFP R2
BRG1
EWSR1-FLI1
EWSR1
FUS

Figure 3A Replicates

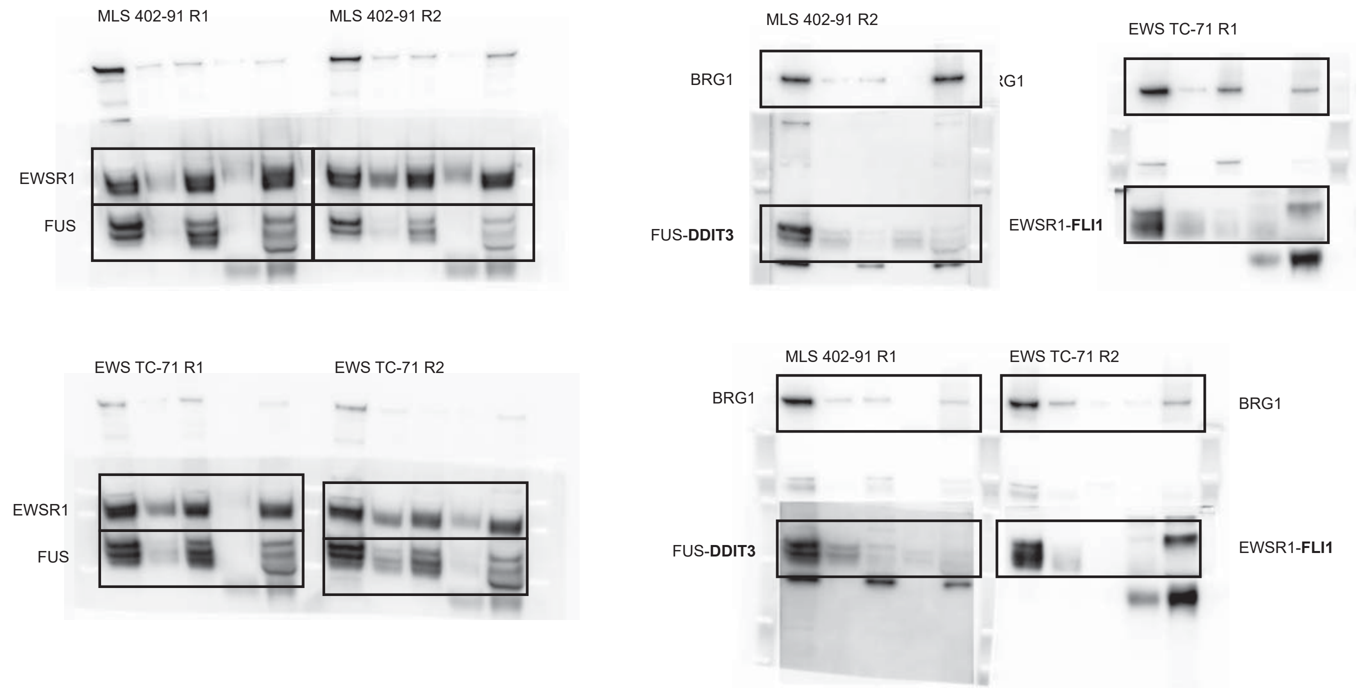

Figure 3C Replicates

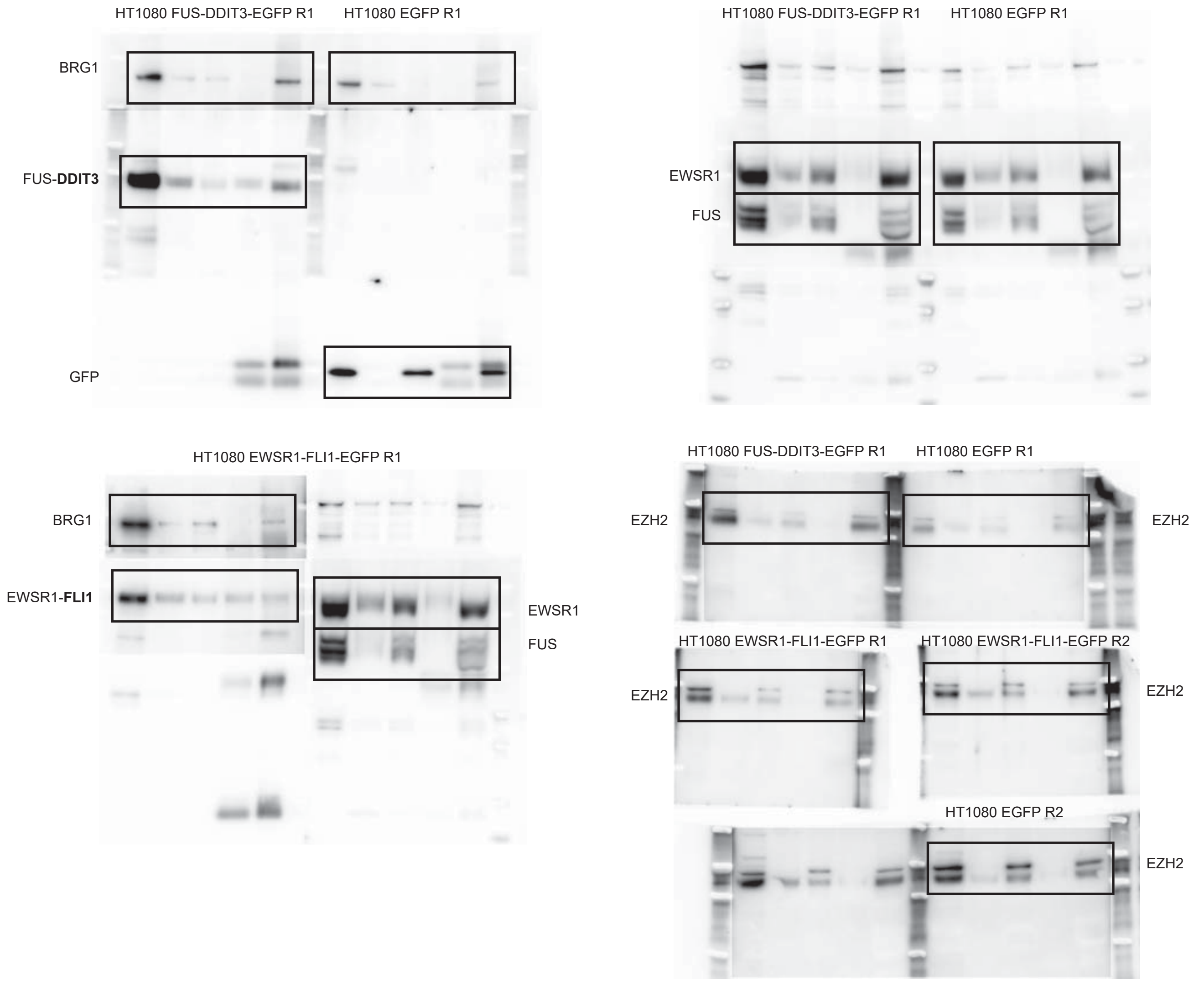

Figure 3C Replicates

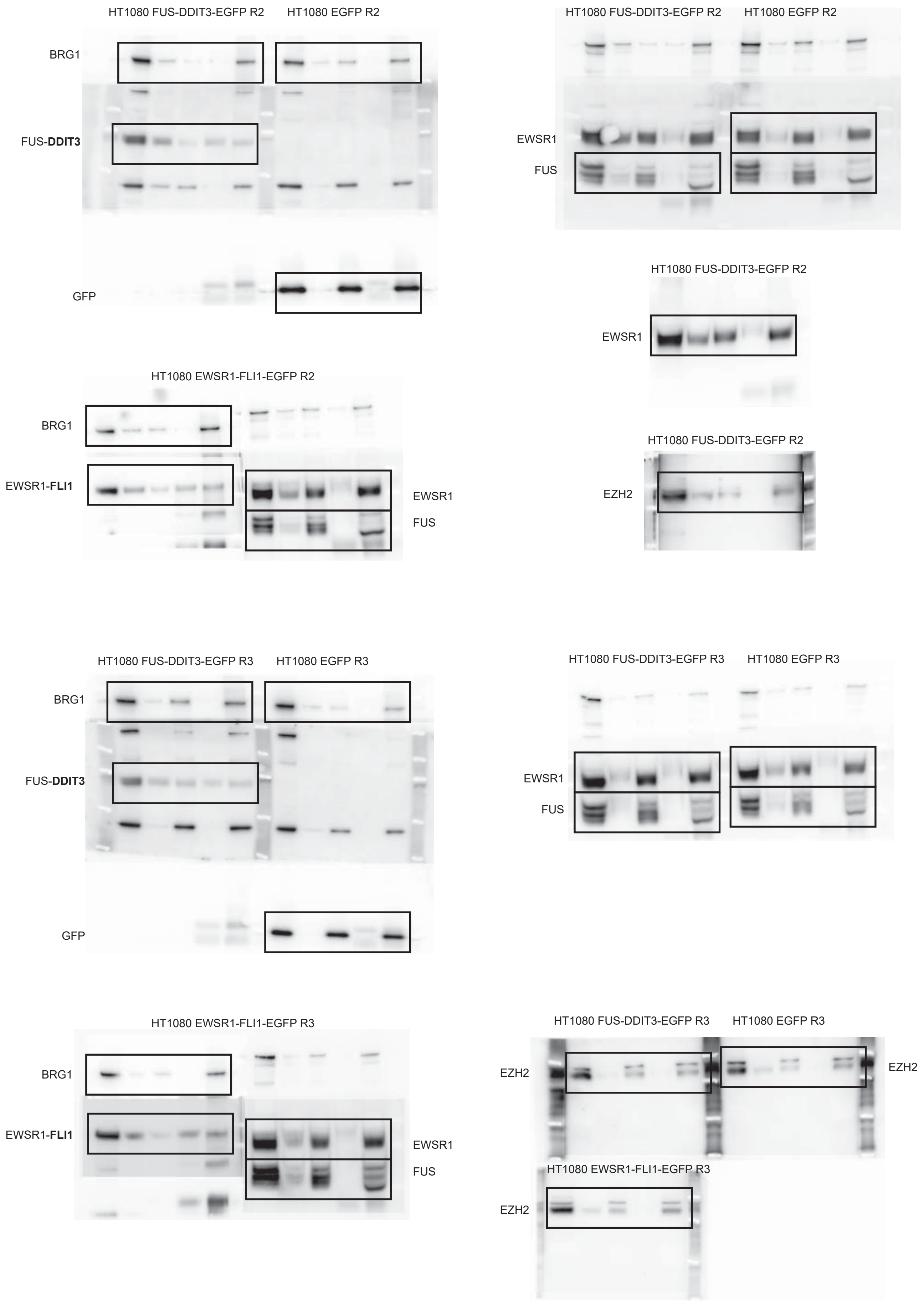

Supplement: Supplementary file 9 — Source Data for Figure 3 [file EMBR-20-e45766-s007.zip › EMBOR-2018-45766_SourceDataForFigure3/EMBOR-2018-45766_SourceDataForFigure3A,C.pdf]
